# Baicalin attenuates PD-1/PD-L1 axis-induced immunosuppression in piglets challenged with *Glaesserella parasuis* by inhibiting the PI3K/Akt/mTOR and RAS/MEK/ERK signalling pathways

**DOI:** 10.1186/s13567-024-01355-1

**Published:** 2024-07-29

**Authors:** Shulin Fu, Jingyang Li, Jiarui You, Siyu Liu, Qiaoli Dong, Yunjian Fu, Ronghui Luo, Yamin Sun, Xinyue Tian, Wei Liu, Jingyi Zhang, Yu Ding, Yitian Zhang, Wutao Wang, Ling Guo, Yinsheng Qiu

**Affiliations:** 1 Hubei Key Laboratory of Animal Nutrition and Feed Science, Wuhan Polytechnic University, Wuhan, 430023 China; 2 Hubei Collaborative Innovation Center for Animal Nutrition and Feed Safety, Wuhan, 430023 China; 3 School of Animal Science and Nutritional Engineering, Wuhan Polytechnic University, Wuhan, 430023 China

**Keywords:** *Glaesserella parasuis*, baicalin, levamisole, BMS-1, immunosuppression, PD-1/PD-L1, PI3K/Akt/mTOR, RAS/MEK/ERK

## Abstract

Infection of piglets with *Glaesserella parasuis* (*G. parasuis*) induces host immunosuppression. However, the mechanism underlying the immunosuppression of piglets remains unclear. Activation of the PD-1/PD-L1 axis has been shown to trigger host immunosuppression. Baicalin possesses anti-inflammatory and immunomodulatory functions. However, whether baicalin inhibits PD-1/PD-L1 activation and thus alleviates host immunosuppression has not been investigated. In this study, the effect of baicalin on the attenuation of piglet immunosuppression induced by *G. parasuis* was evaluated. Seventy piglets were randomly divided into the control group, infection group, levamisole group, BMS-1 group, 25 mg/kg baicalin group, 50 mg/kg baicalin group and 100 mg/kg baicalin group. Following pretreatment with levamisole, BMS-1 or baicalin, the piglets were challenged with 1 × 10^8^ CFU of *G. parasuis*. Our results showed that baicalin, levamisole and BMS-1 modified routine blood indicators and biochemical parameters; downregulated IL-1β, IL-10, IL-18, TNF-α and IFN-γ mRNA expression; and upregulated IL-2 and IL-8 mRNA expression in blood. Baicalin, levamisole and BMS-1 increased the proportions of CD3^+^ T cells, CD3^+^CD4^+^ T cells, CD3^+^CD8^+^ T cells and CD3^–^CD21^+^ B cells in the splenocyte population, increased the proportions of CD3^+^ T cells, CD3^+^CD4^+^ T cells and CD3^+^CD8^+^ T cells in the blood, and inhibited PD-1/PD-L1 and TIM-3 activation. Baicalin, levamisole and BMS-1 reduced p-PI3K, p-Akt, and p-mTOR expression, the p-MEK1/2/MEK1/2 and p-ERK1/2/ERK1/2 ratios and increased RAS expression. Baicalin, levamisole and BMS-1 provided substantial protection against *G. parasuis* challenge and relieved tissue histopathological damage. Our findings might provide new strategies for controlling *G. parasuis* infection and other immunosuppressive diseases.

**Supplementary Information:**

The online version contains supplementary material available at 10.1186/s13567-024-01355-1.

## Introduction

*Glaesserella parasuis* (*G. parasuis*) is a gram-negative bacterial species that causes Glässer’s disease in pigs [1]. The typical characteristics of this disease are fibrinous polyserositis, polyarthritis and meningitis [2]. *G. parasuis* has become one of the most important upper respiratory tract bacterial pathogens of pigs [3]. Infection can cause high morbidity and mortality, leading to serious economic losses to pig farms [4]. To date, 15 serovars of *G. parasuis* have been categorised based on their capsular polysaccharide, but up to 20% of the isolates are not typeable or categorised [5]. Serotypes 4, 5 and 13 are the dominant epidemic strains and are considered to be highly virulent strains [6]. Given the economic losses caused by *G. parasuis*, it is urgent to find a way to control Glässer’s disease.

*Glaesserella parasuis* infection in pigs could lead to host immunosuppression [7]. However, the exact mechanism underlying immunosuppression is still unclear. Innate immune cells play important roles in host defence against pathogen infection by mediating pathogen clearance and regulating the adaptive immune response [8]. Dysregulation of the innate immune system could result in immunosuppression [9]. Previous research has shown that programmed cell death receptor 1 (PD-1) contributes to immunosuppression in sepsis; this phenomenon is related to the upregulation of programmed cell death ligand 1 (PD-L1) in lymphocytes [10]. A decrease in the lymphocyte count leads to changes in T-cell subtypes as well as B-cell dysfunction [11]. Previous reports have shown that the PD-1/PD-L1 axis can activate the phosphoinositide 3-kinase (PI3K)/Akt/mammalian target of rapamycin (mTOR) and RAS/mitogen-activated protein kinase kinase (MEK)/extracellular signal-regulated kinase (ERK) signalling pathways, resulting in immunosuppression in autoimmune diseases [12, 13]. However, the pathways responsible for *G. parasuis*-mediated immunosuppression have not yet been fully described. Elucidation of these pathways could provide a way to control *G. parasuis* infections on pig farms.

Due to the excessive use of antibiotics during animal production, bacterial resistance is becoming increasingly prominent [14]. Traditional Chinese veterinary medicine is considered a promising choice for controlling inflammatory diseases in animal husbandry [15]. Baicalin is the active ingredient of *Scutellaria baicalensis* Georgi 1775 and has important biological functions [16]. Baicalin alleviates gentamicin-induced acute kidney injury via the nuclear factor kappa B (NF-κB) signalling pathway [17]. Moreover, it protects against ulcerative colitis by modulating the gut microbiota–bile acid axis [18]. Baicalin reduces renal fibrosis in 5/6Nx rats by inhibiting the PI3K/Akt/NF-κB signalling pathway [19]. Baicalin enhances proliferation and reduces inflammatory-oxidative stress in hydrogen peroxide (H_2_O_2_)-induced granulosa cell apoptosis via USP48 protein regulation [20]. Our previous study showed that baicalin inhibits PANX-1/P2Y6 signalling pathway activation in porcine aortic vascular endothelial cells infected by *G. parasuis* [21]. Baicalin alleviates G. *parasuis*-induced apoptosis via the PKC-MAPK pathway in porcine peritoneal mesothelial cells [22]. However, whether baicalin can alleviate *G. parasuis*-mediated immunosuppression has not been investigated.

In this study, the ability of baicalin to relieve G. *parasuis*-mediated immunosuppression was explored in a piglet model. Our results might provide novel targets for preventing and controlling *G. parasuis* infection and other immunosuppressive diseases.

## Materials and methods

### Ethics statement

The animal studies were approved by the Animal Care and Use Committee of Wuhan Polytechnic University, Hubei Province, China (WPU202308003). All the experimental animals were euthanised at the end of the experiment.

### Bacteria and growth conditions

*Glaesserella parasuis* SH0165 (serovar 5), a highly virulent strain, was isolated from the lungs of a commercially raised pig that presented with arthritis, fibrinous polyserositis, haemorrhagic pneumonia and meningitis [23]. The SH0165 strain was cultured on tryptic soy agar (TSA) (Difco Laboratories, USA) or in tryptic soy broth (TSB) (Difco Laboratories) supplemented with 10 μg/mL NAD (Guangzhou Saiguo Biotech Co., Ltd., Guangzhou, China) and 10% foetal bovine serum (Sijiqing, Hangzhou, China) at 37 °C.

### Drugs

Baicalin was purchased from Sichuan Xieli Pharmaceutical Co., Ltd. (Pengzhou, Sichuan, China). Levamisole was procured from Beijing Solarbio Science & Technology Co., Ltd. (Beijing, China). BMS-1 was obtained from Shanghai Yuanye Bio-Technology Co., Ltd. (Shanghai, China).

### Experimental design

**Figure 1:**
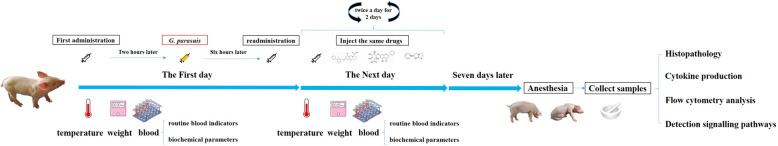
**Schematic design of the study.**

Seventy 30-day-old naturally farrowed early-weaned piglets (Duroc × Landrace × Large White; 9–10 kg in weight) were purchased from the Wuhan Fenglongxin Breeding Professional Cooperative (Wuhan, Hubei, China). The piglets were randomly divided into seven groups: control, infection, levamisole, BMS-1, 25 mg/kg baicalin, 50 mg/kg baicalin and 100 mg/kg baicalin. Before *G. parasuis* challenge, all piglets except those in the control or infection groups were pretreated with an intramuscular injection of the appropriate compound: 15 mg/kg body weight (BW) levamisole, 200 μg/kg BW BMS-1, 25 mg/kg BW baicalin, 50 mg/kg BW baicalin or 100 mg/kg BW baicalin. Two hours after administration on the first day, all piglets, except those in the control group, were intraperitoneally challenged with 1 × 10^8^ colony-forming units (CFU) of *G. parasuis* in 1 mL of TSB. The control group received an equivalent volume of TSB without *G. parasuis* via intraperitoneal injection. After challenge with *G. parasuis* for 6 h, all the groups except the control group were injected with the same drugs. Subsequently, each treatment was administered twice a day for 2 days. The piglets were monitored for 7 days after *G. parasuis* challenge, and their temperature, BW and survival rate were recorded. The scheme of the study design is displayed in Figure 1.

### Detection of routine blood indicators and biochemical parameters

At 24, 48 and 72 h after *G. parasuis* challenge, blood was collected to determine routine blood indicators and biochemical parameters as described previously [24]. The samples were analysed by using commercially available kits (Shanghai Kehua Bio-Engineering Co., Ltd., Shanghai, China) with an automatic blood analyser (Hitachi HITEC 7100, Japan). The routine blood indicators included white blood cell (WBC), red blood cell (RBC), haemoglobin (HGB), platelet (PLT), neutrophil (NE), lymphocyte (LYM), monocyte (MON) and eosinophil (EOS) levels. The biochemical parameters included total bilirubin (T-Bil), total phosphorus (TP), albumin (ALB), aspartate aminotransferase (AST), alanine aminotransferase (ALT), alkaline phosphatase (ALP), total calcium (TC), triglyceride (TG), glucose (GLU), calcium (Ca), inorganic phosphorus (IP), creatinine (CRE), high-density lipoprotein cholesterol (HDL-C), low-density lipoprotein cholesterol (LDL-C), uric acid (UA), blood urea nitrogen (BUN), γ-glutamyl transpeptidase (γ-GT), creatine kinase (CK) and lactate dehydrogenase (LDH).

### Determination of the effects of baicalin, levamisole and BMS-1 on T and B-cell differentiation in the blood and spleen of *G. parasuis*-challenged piglets by flow cytometry

To determine the effect of baicalin, levamisole and BMS-1 on T and B-cell differentiation, blood was collected 24 and 48 h after *G. parasuis* challenge, and spleens were collected 72 h after *G. parasuis* challenge. The spleens were used to prepare splenocytes [25]. Briefly, splenocyte suspensions were acquired by gently passing crushed spleen tissue through 40-μm nylon filters (Merck, USA). The tissue was incubated with lysis solution (8.02% NH_4_Cl, 0.85% NaHCO_3_ and 0.37% ethylenediaminetetraacetic acid [EDTA]) on ice to remove RBCs. After washing three times with phosphate-buffered saline (PBS) and filtering with a 40-μm nylon filter, the splenocytes were resuspended in complete RPMI medium (Gibco, USA) for flow cytometry analysis. Staining and gating strategies were used to identify splenocyte populations and blood cell populations (Additional file 1). Viable cells were then gated for splenocytes and blood cells from the control cells based on FSC and SSC properties. Cells were gated on FSC-Height and FSC-Area. Following staining with mouse anti-porcine CD3ε-FITC (Cat. No. 4510-02, Monoclonal), mouse anti-porcine CD4-SPRD (Cat. No. 4515-13, Monoclonal), mouse anti-porcine CD8a-PE (Cat. No. 4520-09, Monoclonal) and mouse anti-porcine CD21-PE (Cat. No. 4530-09, Monoclonal) (all from SouthernBiotech, Birmingham, AL, USA) to gate for CD3 T cells, CD4 T cells, CD8 T cells and CD21 B cells, the cells were stained with CD3ε-FITC-CD4-SPRD, CD3ε-FITC-CD8a-PE or CD3ε-FITC-CD21-PE to generate a four-quadrant gate. The populations of CD3^+^ T cells, CD3^+^CD4^+^ T cells, CD3^+^CD8^+^ T cells, and CD3^−^CD21^+^ B cells were analysed by using CytExpert SRT software.

### Cytokine production determination by RT-PCR

Blood was collected 48 h after *G. parasuis* challenge, and the production of the cytokines interleukin 1 beta (IL-1β), IL-2, IL-8, IL-10, IL-18, tumour necrosis factor alpha (TNF-α) and interferon gamma (IFN-γ) was determined via reverse transcription-polymerase chain reaction (RT-PCR) [26]. Briefly, total RNA was isolated from the blood with TRIzol reagent (Invitrogen, USA). The RNA concentration and quality were analysed with a Qubit 2.0 fluorometer (Thermo Fisher Scientific, USA). The RNA was reverse transcribed into complementary DNA (cDNA) with reverse transcriptase (TaKaRa, Dalian, China). A SYBR Green PCR Kit (TaKaRa) was used for PCR following the manufacturer’s protocol. The transcription of each sample was repeated at least three times, and GAPDH was used as the internal control. The relative gene expression levels were measured by using the threshold cycle (CT) method. The fold changes in relative gene expression were determined by using the 2^−ΔΔCT^ CT formula. Table 1 lists the RT-PCR primers used [7].

**Table 1:** **Primer sequences for qRT-PCR analysis**

Gene		Nucleotide sequence (5′–3′)	Tm (℃)	Length (bp)
IL-1β	Forward	TCTGCATGAGCTTTGTGCAAG	59.73	155
	Reverse	ACAGGGCAGACTCGAATTCAAC	60.87	
IL-2	Forward	AGCCATTGCTGCTGGATTT	55.05	107
	Reverse	AGCCTGCTTGGGCATGTAA	57.34	
IL-8	Forward	ACAGCAGTAACAACAACAAG	50.18	117
	Reverse	GACCAGCACAGGAATGAG	53.17	
IL-10	Forward	CGTGGAGGAGGTGAAGAGTG	55.40	178
	Reverse	TTAGTAGAGTCGTCATCCTGGAAG	55.60	
IL-18	Forward	AGTAACCATATCTGTGCAGTGT	53.95	155
	Reverse	TCTTATCACCATGTCCAGGAAC	53.04	
TNF-α	Forward	CGCTCTTCTGCCTACTGCACTTC	60.68	164
	Reverse	CTGTCCCTCGGCTTTGACATT	57.77	
IFN-γ	Forward	GAGGTTCCTAAATGGTAGCTCTGG	57.08	164
	Reverse	TCTGACTTCTCTTCCGCTTTCTT	55.55	
PD-1	Forward	GCGGAATGTCAAGGAAACC	54.31	150
	Reverse	CTGTACCCGTGGAGGAGGA	59.14	
PD-L1	Forward	AATGGCGAGGAAGACCTGAA	56.24	137
	Reverse	CAGCAGTAAACCCCTGCATCT	57.62	
TIM-3	Forward	TCAAGCCTCATCACTTTGG	53.70	145
	Reverse	TGACGGAGCAGTAACACTC	50.80	
PI3K	Forward	TTGCTACAATCAATCGCCAGGAGAC	59.32	147
	Reverse	CTTCCCGTTGTTGCCATCGTTTG	59.67	
Akt	Forward	GGACGGGCACATCAAGATCACTG	60.84	126
	Reverse	TAGTCGTTGTCCTCCAGCACCTC	61.16	
mTOR	Forward	AGTACCTCCAGGACACCATGAACC	60.88	108
	Reverse	CAGACCTCACAGCCACAGAAAGC	60.97	
GAPDH	Forward	GGCACAGTCAAGGCGGAGAAC	61.89	105
	Reverse	AGCACCAGCATCACCCCATTTG	60.99	

The sequences of the primers used for qRT-PCR, except for TIM-3, were previously described [7]. The TIM-3 primer sequence was used in this study.

### Detection of the effects of baicalin, levamisole and BMS-1 on the activation of PD-1/PD-L1, PI3K/Akt/mTOR and RAS/MEK/ERK signalling in the aorta of *G. parasuis*-challenged piglets by western blotting

The effects of baicalin, levamisole and BMS-1 on the activation of the PD-1/PD-L1, PI3K/Akt/mTOR and RAS/MEK/ERK signalling pathways were explored by western blotting, as described previously with some minor modifications [27]. Briefly, protein was extracted from the aorta by using a total protein extraction kit (Beyotime Biotechnology, Shanghai, China) and then subjected to separation via sodium dodecyl sulphate-polyacrylamide gel electrophoresis (SDS-PAGE) with a 12% gel. The separated protein was transferred to polyvinylidene difluoride (PVDF) membranes, which were incubated with 5% non-fat milk at 37 °C for 1 h. After washing three times with TBST, the membranes were incubated with primary antibody of PD-1 (Cat. No. A23007, Monoclonal, 1:500, ABclonal), PD-L1 (Cat. No. 66248-1, Monoclonal, 1:2000, proteintech), PI3K (Cat. No. A4992, Monoclonal, 1:500, ABclonal), p-PI3K (Cat. No. AP0427, Polyclonal, 1:1000, ABclonal), Akt (Cat. No. 10176-2, Polyclonal, 1:2000, proteintech), p-Akt (Cat. No. 66444-1, Monoclonal, 1:2000, proteintech), mTOR (Cat. No. 66888-1, Monoclonal, 1:5000, proteintech), p-mTOR (Cat. No. 67778-1, Monoclonal, 1:2000, proteintech), RAS (Cat. No. A19779, Monoclonal, 1:1000, ABclonal), MEK1/2 (Cat. No. A4868, Monoclonal, 1:6000, ABclonal), p-MEK1/2 (Cat. No. AP1349, Monoclonal, 1:10 000, AB clonal), ERK1/2(Cat. No. A22447, Monoclonal, 1:5000, ABclonal), p-ERK1/2 (Cat. No. AP0485, Monoclonal, 1:2000, ABclonal), or GAPDH (Cat. No. 10494-1-AP, Polyclonal, 1:5000, proteintech) at 4 °C for 12 h, respectively. The membranes were washed three times with TBST and then incubated with the corresponding HRP-conjugated goat anti-mouse IgG (H+L) (PD-1, PD-L1, p-Akt, mTOR, p-mTOR, RAS) (ABClonal) or HRP-conjugated goat anti-rabbit IgG (PI3K, p-PI3K, Akt, RAS, MEK1/2, p-MEK1/2, ERK1/2, p-ERK1/2, GAPDH) (Abbkine) at 37 °C for 1 h. The protein bands were visualised by using an enhanced chemiluminescence (ECL) kit (ABclonal). The coloured bands were analysed by using ImageJ software to measure the gray values with a FluorChemFC2 AIC system (Alpha Innotech, USA). The protein expression levels were determined by comparing the gray values of the colored bands with the gray values of the internal controls.

### Histopathological analysis by haematoxylin–eosin staining

The lung lesion score was determined as described by Fu et al. [28]. The spleen, lung and brain were removed, fixed by immersion in 10% neutral buffered formalin and then embedded in paraffin. Four-micrometre tissue sections were cut and stained with haematoxylin and eosin (HE) following a standard protocol [29]. The stained sections were examined under a light microscope (Olympus BX43, Tokyo, Japan).

### Statistical analysis

The experimental data are expressed as the mean ± standard deviation. Statistical differences between two groups were estimated with one-way analysis of variance (ANOVA) using the statistical package IBM SPSS Statistics software (SPSS). The log-rank test was used for survival analysis. *P* < 0.05 indicates a statistically significant difference. ^##^*P* < 0.01 versus controls; ^###^*P* < 0.001 versus controls; *significance at *P* < 0.05; **significance at *P* < 0.01; ***significance at *P* < 0.001.

## Results

### Baicalin, levamisole and BMS-1 attenuated the mortality of *G. parasuis*-challenged piglets

**Figure 2:**
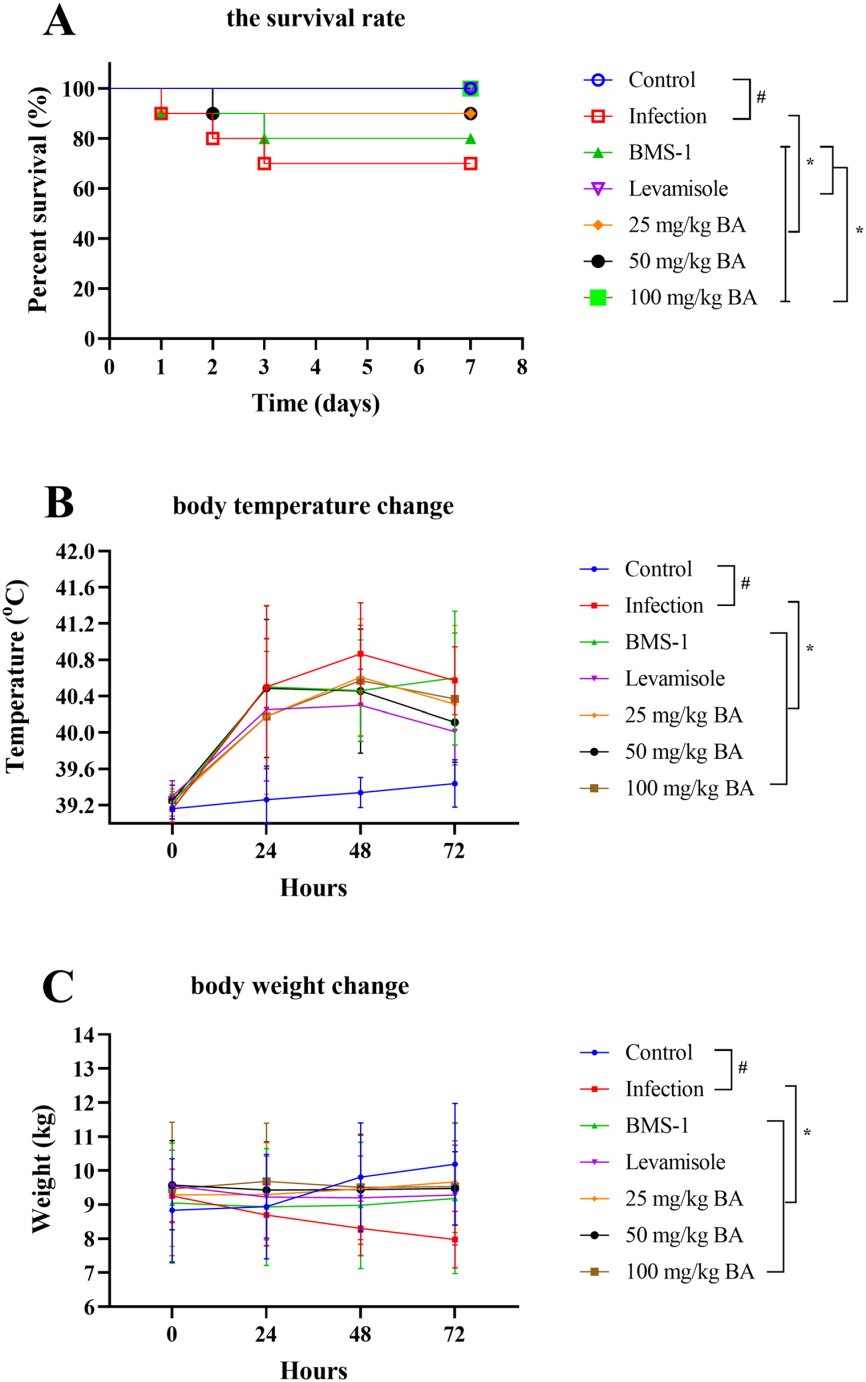
**Baicalin, levamisole, and BMS-1 provided protection against G. parasuis challenge.** The piglets were injected with baicalin, levamisole, or BMS-1 and were challenged with *G. parasuis*. The survival rate, body temperature change, and body weight change were recorded. **A** Survival rate; **B** body temperature change; **C** body weight change; BA: baicalin; ^#^*P* < 0.05 versus controls; *significance at *P* < 0.05.

The survival rate of the *G. parasuis*-challenged piglets was significantly lower than that of the control piglets (*P* < 0.05; Figure 2A). When the piglets were pretreated with baicalin, levamisole or BMS-1, the survival rate increased (*P* < 0.05). In addition, the baicalin groups had a greater survival rate than did the BMS-1 and levamisole groups (*P* < 0.05).

Forty-eight hours after *G. parasuis* challenge, the infection group had a significantly greater temperature than the control group (*P* < 0.05; Figure 2B). Compared to those in the infection group, baicalin, levamisole and BMS-1 reduced the increase in body temperature (*P* < 0.05). Seventy-two hours after *G. parasuis* challenge, the BW of the infected group decreased compared to that of the control group (*P* < 0.05; Figure 2C). Compared with those in the infection group, baicalin, levamisole and BMS-1 alleviated this decrease in BW (*P* < 0.05).

### Baicalin, levamisole and BMS-1 improved the routine blood indicators and biochemical parameters of *G. parasuis*-challenged piglets

**Figure 3:**
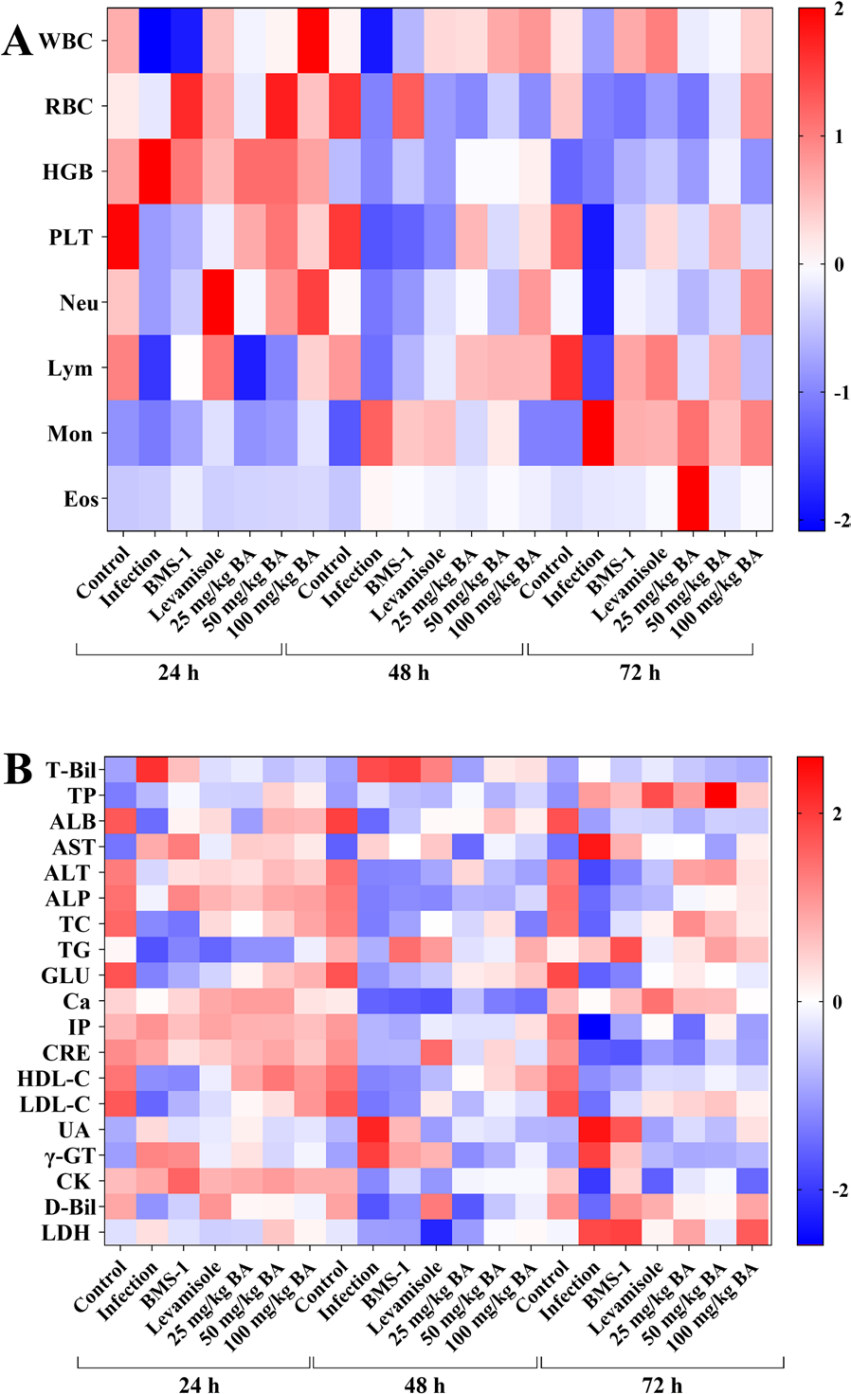
**Routine blood tests and blood biochemical parameters.** At 24, 48 and 72 h after *G. parasuis* challenge, blood was collected to determine routine blood indicators and biochemical parameters. Heatmaps were generated to visualize Z scores using routine blood test data and blood biochemical parameters. **A** routine blood test; **B** blood biochemical parameters; BA: baicalin.

As shown in Figure 3A, 24 h after *G. parasuis* challenge, WBC, PLT and LYM were decreased, and HGB was increased in the infection group (*P* < 0.05). The WBC and LYM counts in the levamisole group and the LYM count in the BMS-1 group were greater than those in the infection group (*P* < 0.05). Treatment with 50 or 100 mg/kg baicalin corrected the WBC, PLT, and LYM levels compared to those in the infection group (*P* < 0.05). Forty-eight to seventy-two hours after *G. parasuis* challenge, the RBC, PLT, and LYM levels were decreased, and the MON level was increased in the infection group compared to those in the control group (*P* < 0.05) (Figure 3A). Levamisole increased the WBC and LYM levels, and 25–100 mg/kg baicalin increased the RBC, PLT, and LYM levels compared to those in the infection group (*P* < 0.05) (Figure 3A).

Regarding the blood biochemical parameters, 24–72 h after *G. parasuis* challenge, the levels of ALB, ALT, ALP, TC, GLU, IP, CRE, HDL-C, LDL-C, γ-GT, and CK decreased, and the levels of AST, UA, and BUN increased in the infection group compared to those in the control group (*P* < 0.05; Figure 3B). Compared with infection alone, levamisole and BMS-1 increased ALP, TC, LDL-C, and CK (*P* < 0.05; Figure 3B). Moreover, compared with those in the infection group, the AST, ALP, TC, ALT, TC, GLU, LDL-C, UA and BUN levels in the 25–100 mg/kg baicalin group returned to normal levels (*P* < 0.05; Figure 3B).

### Baicalin, levamisole and BMS-1 modified T and B-cell differentiation in the blood and spleen of *G. parasuis*-challenged piglets

**Figure 4:**
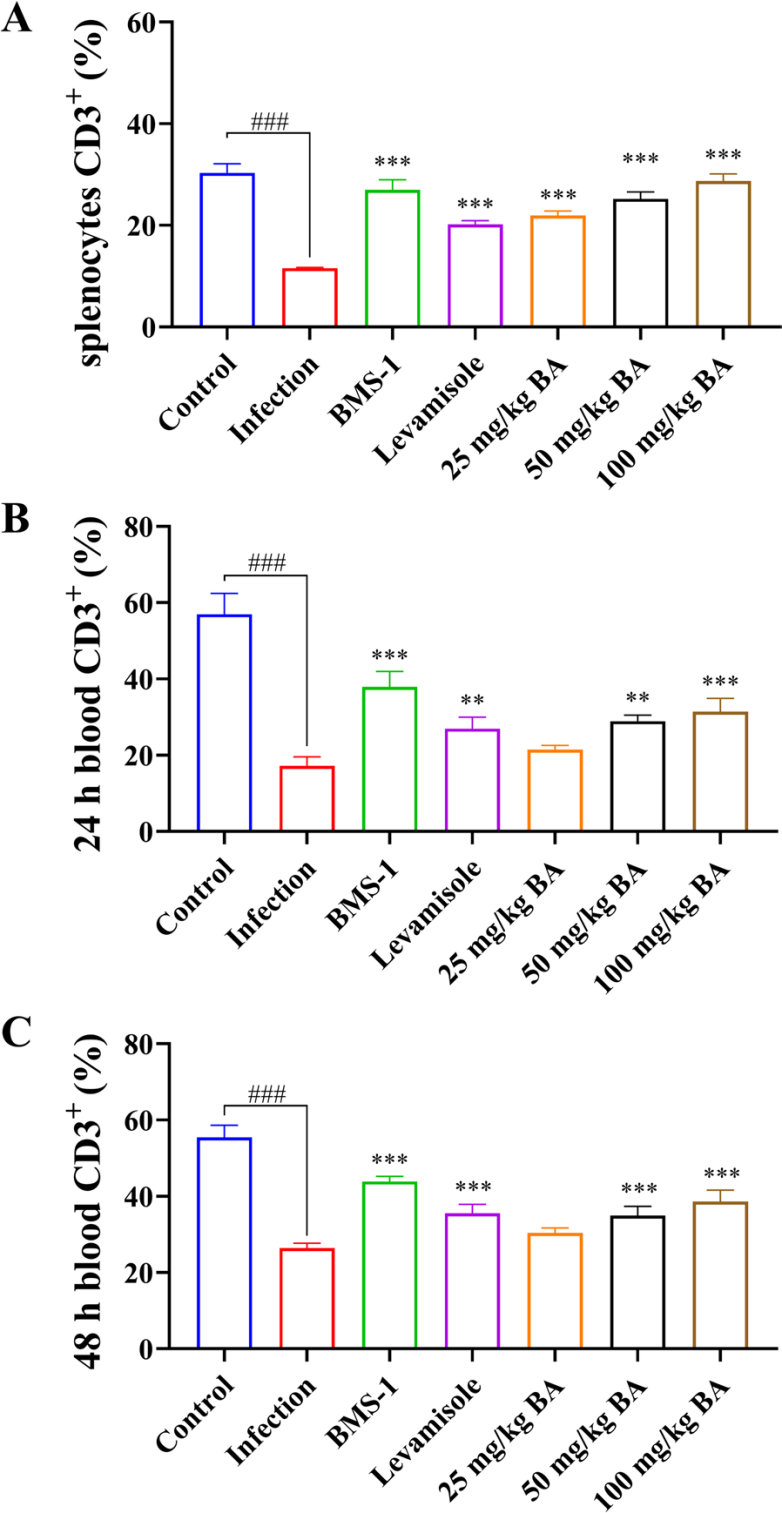
**Measurement of the effect of baicalin, levamisole, and BMS-1 on the proportion of CD3**^**+**^** T cells among splenocytes and blood by flow cytometry.** Blood and spleen were collected and splenocytes were prepared. The proportion of CD3^+^ T cells was determined by flow cytometry. **A** CD3^+^ T-cell proportions of splenocytes; **B** CD3^+^ T-cell proportions of blood after 24 h; **C** CD3^+^ T-cell proportions of blood after 48 h; BA: baicalin; ^###^*P* < 0.001 versus controls; **significance at *P* < 0.01; ***significance at *P* < 0.001.

**Figure 5:**
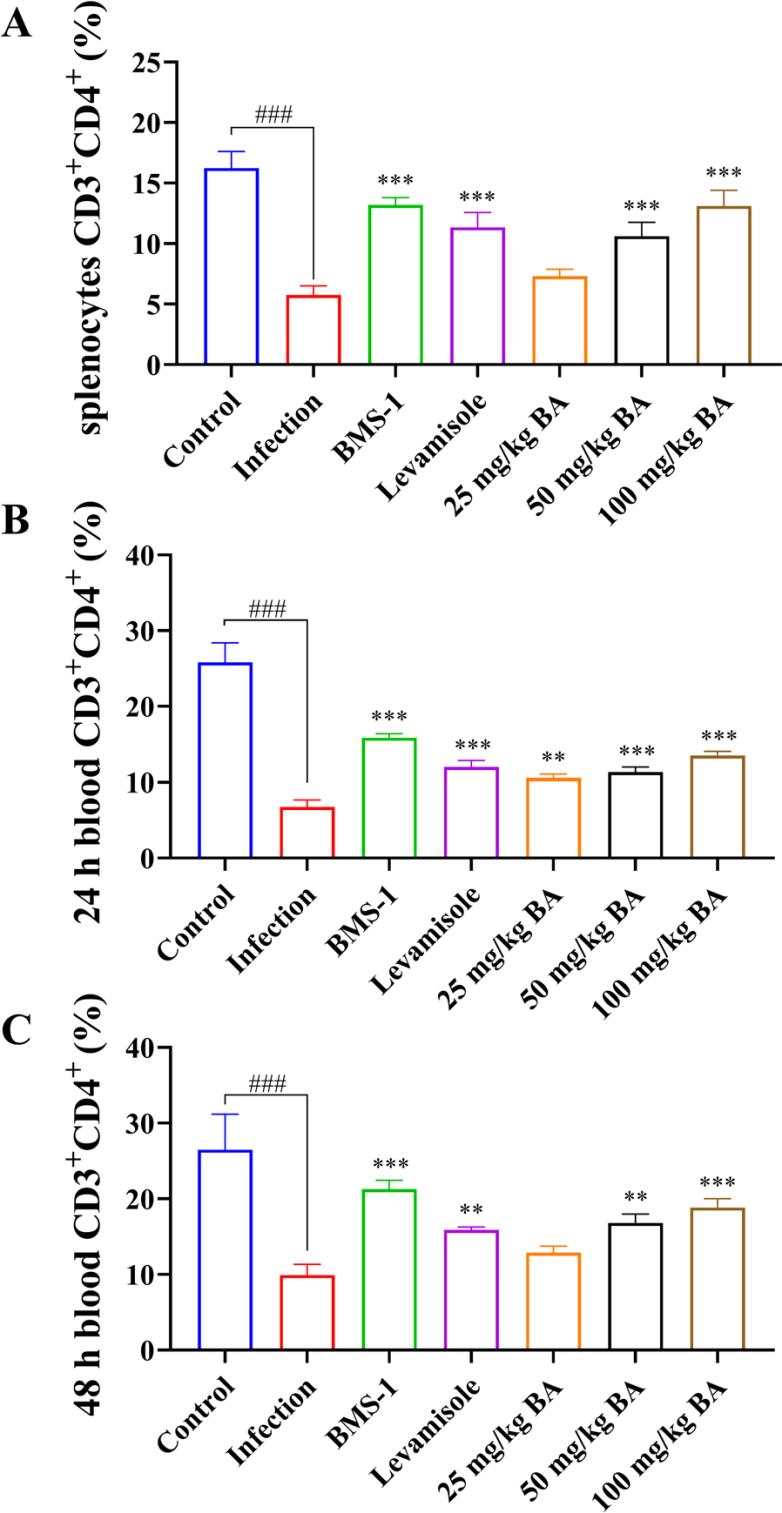
**Measurement of the effect of baicalin, levamisole, and BMS-1 on the proportion of CD3**^**+**^**CD4**^**+**^** T cells in the splenocyte population and blood by flow cytometry.** Blood and spleen were collected and splenocytes were prepared. The proportion of CD3^+^CD4^+^ T cells was determined by flow cytometry. **A** CD3^+^CD4^+^ T-cell proportion in splenocytes; **B** CD3^+^CD4^+^ T-cell proportion in blood after 24 h; **C** CD3^+^CD4^+^ T-cell proportion in blood after 48 h; BA: baicalin; ^###^*P* < 0.001 versus controls; **significance at *P* < 0.01; ***significance at *P* < 0.001.

**Figure 6:**
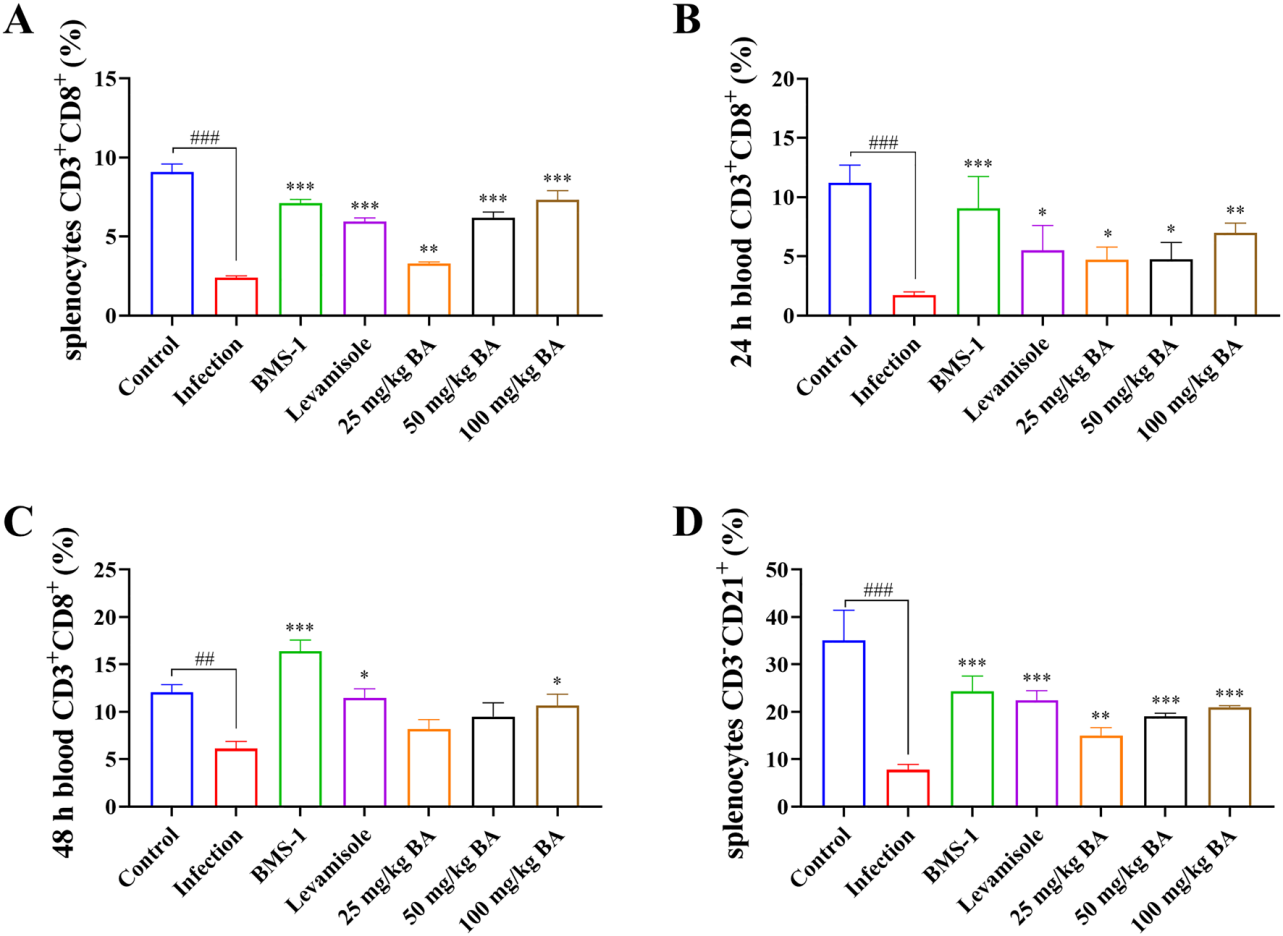
**The effects of baicalin, levamisole, and BMS-1 on the proportions of CD3**^**+**^**CD8**^**+**^** T cells among splenocytes and blood and the proportion of CD3**^**–**^**CD21**^**+**^** B cells among splenocytes were measured by flow cytometry.** Blood was collected, the spleen was collected, and splenocytes were prepared. The proportions of CD3^+^CD8^+^ T cells and CD3^–^CD21^+^ B cells were determined by flow cytometry. **A** Proportion of CD3^+^CD8^+^ T cells among splenocytes; **B** proportion of CD3^+^CD8^+^ T cells among blood cells after 24 h; **C** proportion of CD3^+^CD8^+^ T cells among blood cells after 48 h; **D** proportion of CD3^–^CD21^+^ B cells among splenocytes; BA: Baicalin; ^##^*P* < 0.01 versus controls; ^###^*P* < 0.001 versus controls; *significance at *P* < 0.05; **significance at *P* < 0.01; ***significance at *P* < 0.001.

Seventy-two hours after *G. parasuis* challenge, there was a decrease in the proportions of CD3^+^ T cells, CD3^+^CD4^+^ T cells, and CD3^+^CD8^+^ T cells among splenocytes in the infection group compared to those in the control group (*P* < 0.001); levamisole, BMS-1 and 50 and 100 mg/kg baicalin improved the proportions of CD3^+^ T cells, CD3^+^CD4^+^ T cells and CD3^+^CD8^+^ T cells in the infection group (*P* < 0.05; Figure 4A, Additional file 2, panels A1–A7; Figure 5B, Additional file 3, panels A1–A7; Figure 6A, Additional file 4, panels A1–A7). There was also a decrease in the proportion of CD3^–^CD21^+^ B cells among splenocytes in the infection group compared to that in the control group (*P* < 0.001); levamisole, BMS-1 and 25–100 mg/kg baicalin enhanced the proportion of CD3^–^CD21^+^ B cells compared to that in the infection group (*P* < 0.001; Figure 6D, Additional file 5).

Twenty-four hours after *G. parasuis* challenge, the proportions of CD3^+^ T cells, CD3^+^CD4^+^ T cells and CD3^+^CD8^+^ T cells in the blood were significantly lower in the infection group than in the control group (*P* < 0.05; Figure 4B, Additional file 2, panels B1, B2; Figure 5B, Additional file 3, panels B1, B2; Figure 6B, Additional file 4, panels B1, B2). Compared with those in the infection group, the proportions of CD3^+^ T cells, ^CD4+^ T cells and CD3^+^CD8^+^ T cells among splenocytes in the levamisole and BMS-1 groups were greater (*P* < 0.05) (*P* < 0.05; Figure 4B, Additional file 2, panels B3, B4; Figure 5B, Additional file 3, panels B3, B4; Figure 6B, Additional file 4, panels B3, B4). Moreover, 50 and 100 mg/kg baicalin increased the proportions of CD3^+^ T cells, CD3^+^CD4^+^ T cells and CD3^+^CD8^+^ T cells compared to those in the infection group; these effects were dose dependent (*P* < 0.05; Figure 4B, Additional file 2, panels B6, B7; Figure 5B, Additional file 3, panels B6, B7; Figure 6B, Additional file 4, panels B6, B7). There were similar results 48 h after *G. parasuis* challenge (*P* < 0.05; Figure 4C, Additional file 2, panels C1–C7; Figure 5C, Additional file 3, panels C1–C7; Figure 6C, Additional file 4, panels C1–C7).

### Baicalin, levamisole, and BMS-1 inhibited cytokine production in the blood of *G. parasuis*-challenged piglets

**Figure 7:**
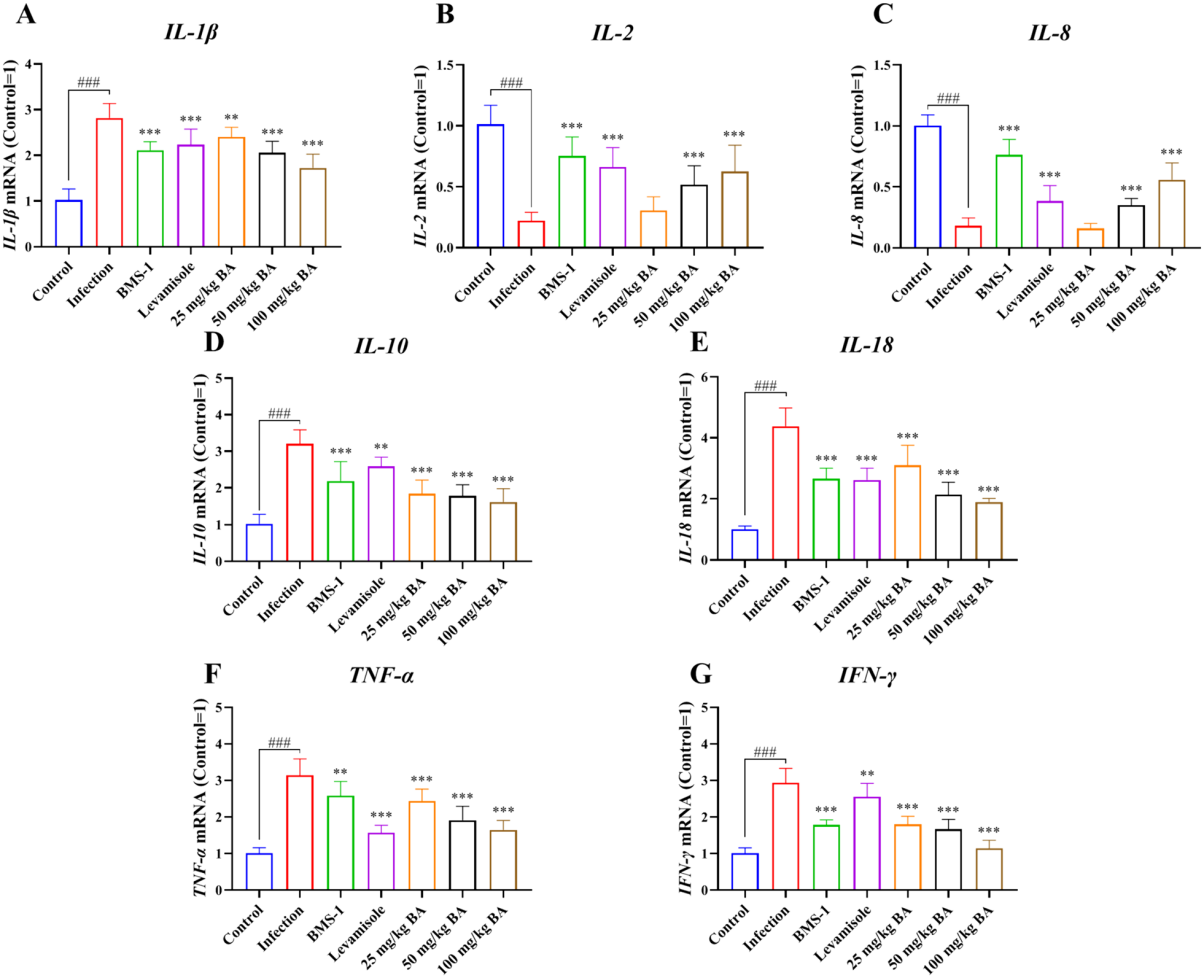
**Determination of the effect of baicalin, levamisole, and BMS-1 on cytokine production in the blood of piglets challenged with** ***G. parasuis*.** Blood was collected, and total RNA was isolated. The cytokine expression levels were determined by RT-PCR. **A** IL-1β; **B** IL-2; **C** IL-8; **D** IL-10; **E** IL-18; **F** TNF-α; **G** IFN-γ; BA: baicalin; ^###^*P* < 0.001 versus controls; **significance at *P* < 0.01; ***significance at *P* < 0.001.

Forty-eight hours after *G. parasuis* challenge, IL-1β, IL-10, IL-18, TNF-α and IFN-γ mRNA expression was increased, and IL-2 and IL-8 mRNA expression was decreased in the infection group compared to the control group (*P* < 0.001; Figure 7). Compared with infection alone, levamisole and BMS-1 reduced IL-1β, IL-10, IL-18, TNF-α and IFN-γ mRNA expression and increased IL-2 and IL-8 mRNA expression (*P* < 0.01). In addition, 25–100 mg/kg baicalin attenuated the changes in IL-1β, IL-10, IL-18, TNF-α and IFN-γ mRNA expression compared to that in the infection group (*P* < 0.01; Figure 7). Finally, 50 and 100 mg/kg baicalin increased IL-2 and IL-8 mRNA expression compared to that in the infection group (*P* < 0.001).

### Baicalin, levamisole and BMS-1 attenuated PD-1/PD-L1 and TIM-3 activation in *G. parasuis*-challenged piglets

**Figure 8:**
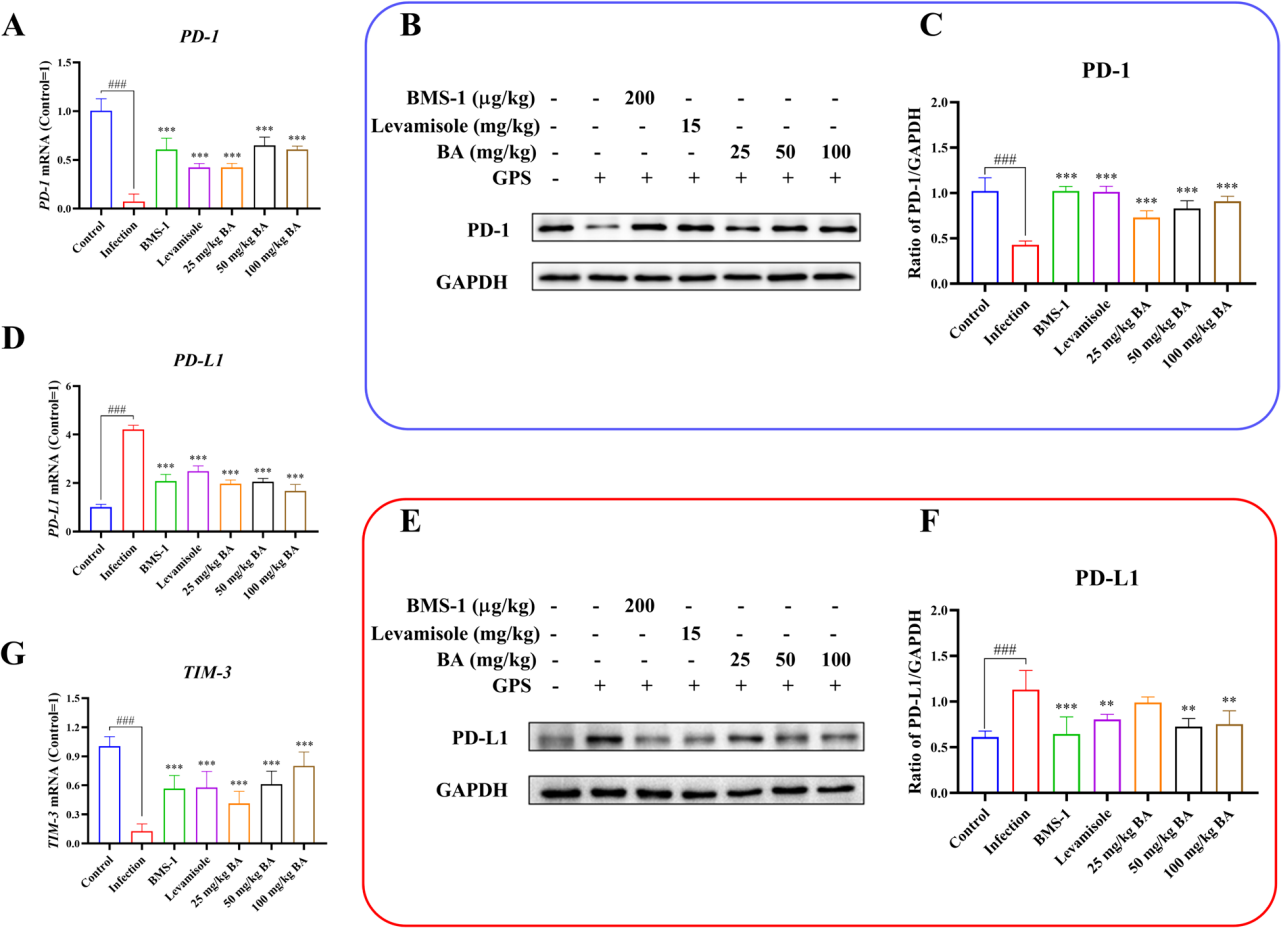
**Determination of the effect of baicalin, levamisole, and BMS-1 on PD-1/PD-L1 and TIM-3 mRNA and protein expression levels in the aorta by RT-PCR and western blot.** **A** PD-1 expression at the mRNA level; **B**, **C** PD-1 expression at the protein level; **D** PD-L1 expression at the mRNA level; **E**, **F** PD-L1 expression at the protein level; **G** TIM-3 expression at the mRNA level; *GPS: G. parasuis*; BA: baicalin; ^###^*P* < 0.001 versus controls; **significance at *P* < 0.01; ***significance at *P* < 0.001.

The PD-1/PD-L1 axis is related to immunosuppression because it inhibits the functions of T cells [30]; thus, the effects of baicalin, levamisole and BMS-1 on PD-1/PD-L1 activation in the aorta were investigated. As shown in Figure 8A, D, G,* G. parasuis* challenge promoted an increase in PD-L1 and TIM-3 mRNA expression and a decrease in PD-1 mRNA expression compared to those in the control group, while levamisole, BMS-1 and baicalin reduced PD-L1 and TIM-3 and upregulated PD-1 mRNA expression compared to those in the infection group (*P* < 0.001). Compared with those in the control group, the PD-1 protein level was decreased, and the PD-L1 protein level was increased in the infection group (*P* < 0.001; Figure 8B, C, E, F). Treatment with 50–100 mg/kg baicalin, levamisole, or BMS-1 upregulated PD-1 protein expression and reduced PD-L1 protein expression compared to those in the infection group (*P* < 0.01; Figure 8B, C, E, F).

### Baicalin, levamisole and BMS-1 weakened PI3K/Akt/mTOR signalling pathway activation in *G. parasuis*-challenged piglets

**Figure 9:**
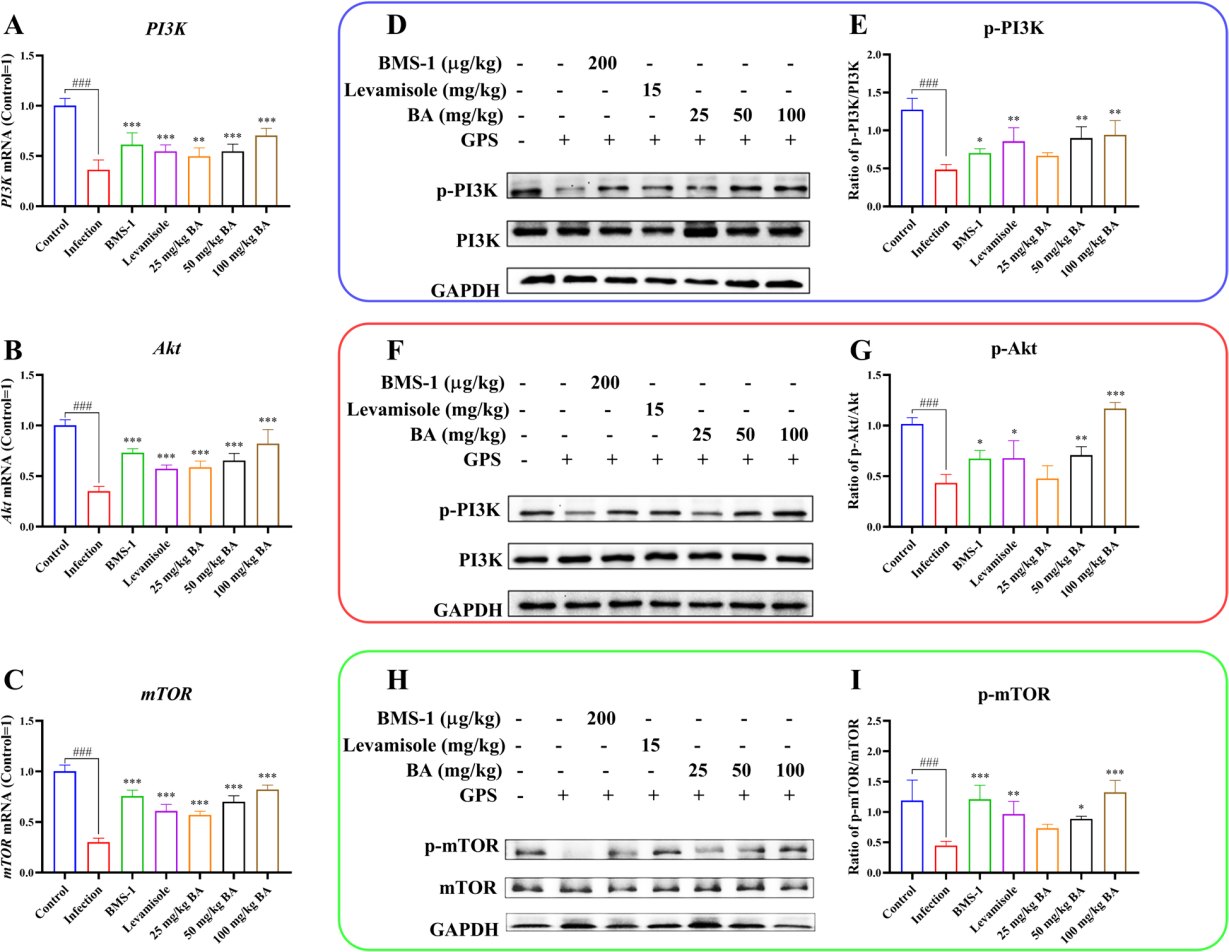
**Measurement of the effect of baicalin, levamisole, and BMS-1 on the PI3K/Akt/mTOR mRNA and protein expression levels in the aorta by RT-PCR and western blotting.** **A** PI3K expression at the mRNA level; **B** Akt expression at the mRNA level; **C** mTOR expression at the mRNA level; **D**, **E** PI3K expression at the protein level; **F**, **G** Akt expression at the protein level; **H**, **I** mTOR expression at the protein level; GPS:* G. parasuis*; BA: baicalin; ^###^*P* < 0.001 versus controls; *significance at *P* < 0.05; **significance at *P* < 0.01; ***significance at *P* < 0.001.

Previous research reported that the PD-1/PD-L1 axis could activate the PI3K/Akt/mTOR signalling pathway, leading to host immunosuppression [31]; thus, the mRNA and protein expression of the relevant components of this pathway in the aorta were evaluated. PI3K, Akt and mTOR mRNA expression was downregulated in the infection group compared to that in the control group, while baicalin, levamisole and BMS-1 increased the expression of these components compared to that in the infection group (*P* < 0.01; Figure 9A–C). p-PI3K, p-Akt and p-mTOR protein expression was lower in the infection group than in the control group (*P* < 0.001; Figure 9D–I), but their expression was greater in the levamisole, BMS-1 and 50–100 mg/kg baicalin groups than in the infection group (*P* < 0.05; Figure 9D–I). These results suggest that baicalin, levamisole and BMS-1 inhibit PI3K/Akt/mTOR pathway activation in *G. parasuis*-challenged piglets.

### Baicalin, levamisole and BMS-1 reduced RAS/MEK/ERK pathway activation in *G. parasuis*-challenged piglets

**Figure 10:**
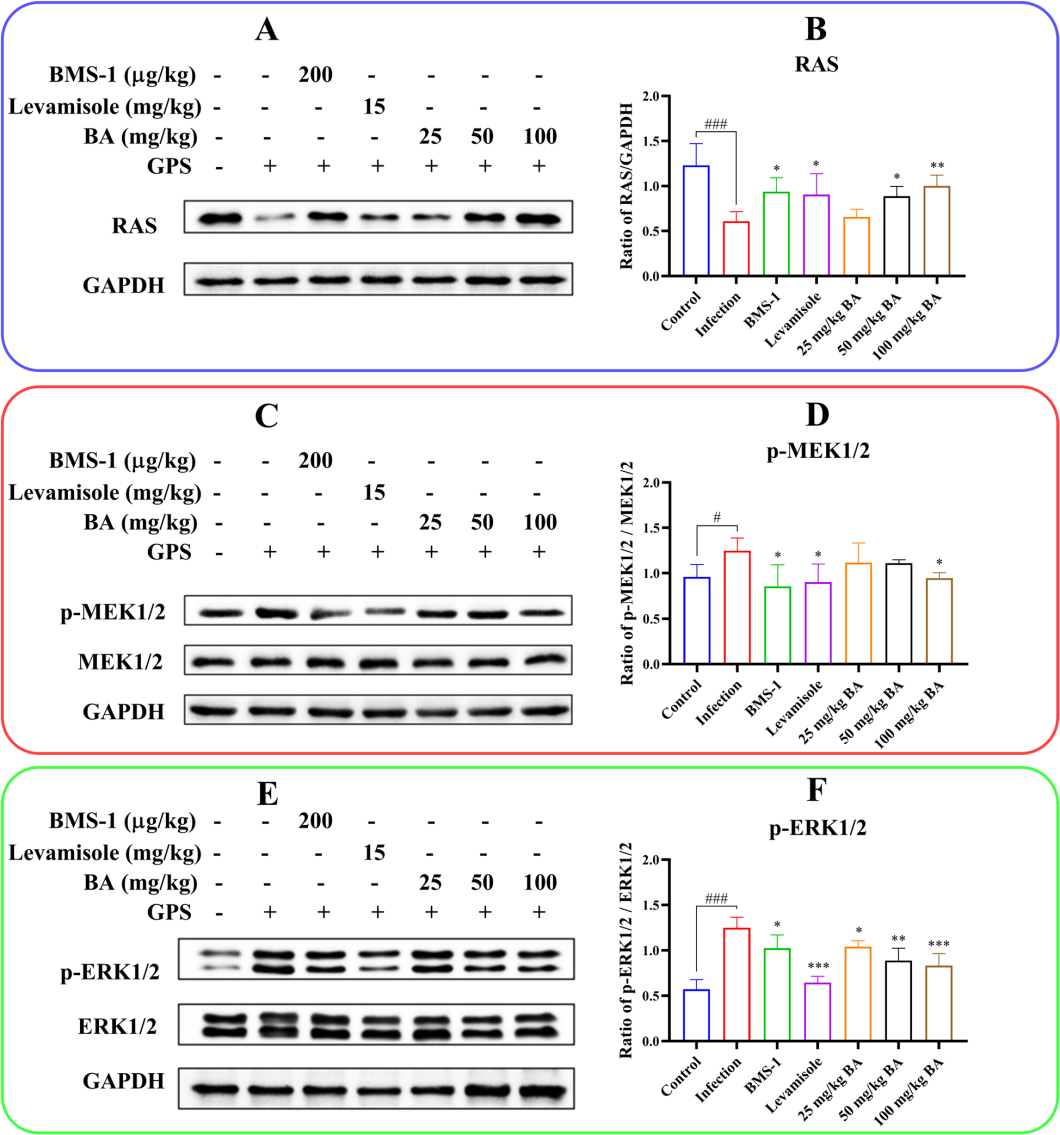
**Evaluation of the effect of baicalin, levamisole, and BMS-1 on RAS/MEK/ERK protein expression levels in the aorta by western blot.** **A**, **B** RAS protein expression; **C**, **D** p-MEK1/2 protein expression; **E**, **F** p-ERK1/2 protein expression; GPS:* G. parasuis*; BA: baicalin; ^#^*P* < 0.05 versus controls; ^###^*P* < 0.001 versus controls; *significance at *P* < 0.05; **significance at *P* < 0.01; ***significance at *P* < 0.001.

The PD-1/PD-L1 axis regulates the RAS/MEK/ERK signalling pathway, which affects cell cycle progression in T lymphocytes, leading to immunosuppression [32]. Thus, the levels of components of the RAS/MEK/ERK pathway in the aorta were investigated by using western blotting. Compared to the control group, the infection group showed decreased RAS protein expression (51.2%) and increased p-MEK1/2/MEK1/2 and p-ERK1/2/ERK1/2 ratios (30.2% and 119.3%, respectively) (*P* < 0.05; Figure 10). However, there was increased RAS expression in the levamisole group (49.2%) and the BMS-1 group (54.1%) (*P* < 0.05; Figure 10). Treatment with 50–100 mg/kg baicalin also upregulated abnormal RAS protein expression and attenuated the p-MEK1/2/MEK1/2 and p-ERK1/2/ERK1/2 ratios compared to those in the infection group (*P* < 0.05; Figure 10). These findings indicate that baicalin, levamisole, and BMS-1 suppress the RAS/MEK/ERK signalling pathway in the aortas of *G. parasuis*-challenged piglets.

### Baicalin, levamisole, and BMS-1 relieved histopathological damage in *G. parasuis*-challenged piglets

**Figure 11:**
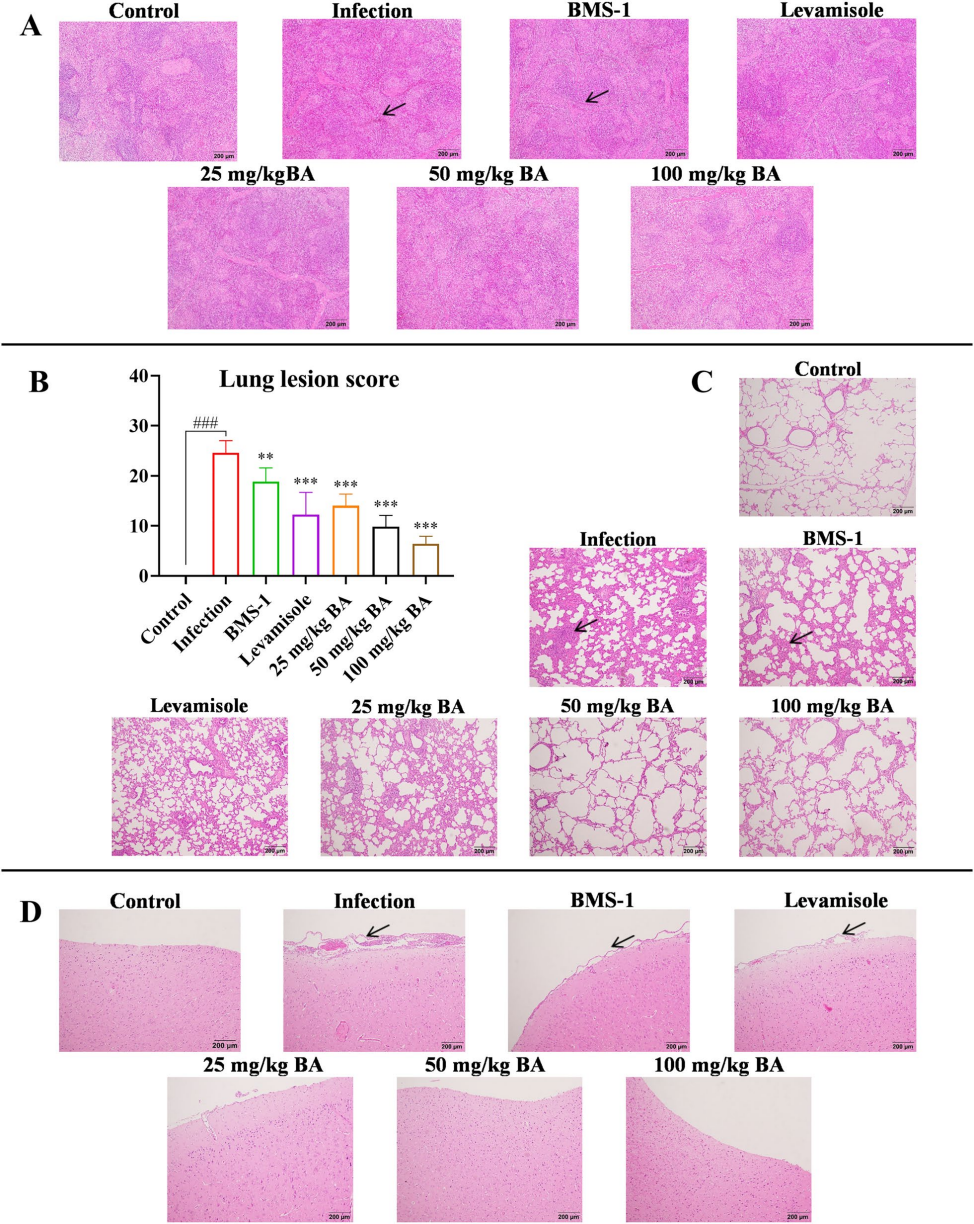
**Detection of the effect of baicalin, levamisole, and BMS-1 on tissue histopathological damage in piglets challenged with** ***G. parasuis*.** Spleen, lung, and brain tissues were collected, and tissue sections were cut and stained with haematoxylin and eosin. **A** spleen; **B** lung lesion score; **C** lung; **D** brain; BA: baicalin. ^###^*P* < 0.001 versus controls; **significance at *P* < 0.01; ***significance at *P* < 0.001.

The animals were monitored after challenge, and lung lesion scores were recorded. The results showed that the number of lung lesions significantly increased in the infection group, while the number of lung lesions significantly decreased in the levamisole, BMS-1 and baicalin groups (*P* < 0.01; Figure 11). The lung, spleen and brain were collected to evaluate microscopic tissue damage. There was no obvious histopathological damage in the control group, while the infection group exhibited oedema, haemorrhage and inflammatory cell infiltration in the lung; inflammatory cell infiltration, red pulp hyperaemia and white pulp haemorrhage in the spleen; and haemorrhage and inflammatory cell infiltration in the brain (Figure 11). However, the levamisole, BMS-1 and baicalin groups showed only minor damage (Figure 11), which suggested that baicalin, levamisole, and BMS-1 could relieve *G. parasuis*-induced histopathological damage.

## Discussion

This study used a piglet infection model to explore how baicalin, levamisole and BMS-1 could relieve host immunosuppression induced by *G. parasuis*. These results are the first indication of how baicalin, levamisole and BMS-1 counteract the immunosuppression elicited by *G. parasuis*.

At 24–72 h after *G. parasuis* challenge, the lymphocyte counts decreased significantly in the infection group compared to those in the control group. A previous study reported that patients with sepsis and low lymphocyte counts are more likely to present with immunosuppression [33]. The low abundance of lymphocytes could be the result of numerical changes in multiple T-cell subtypes [34]. Abnormal proliferation and differentiation of T cells can lead to a weakened ability to clear bacteria, thereby causing immunosuppression [35], which might be an important mechanism leading to host immunosuppression. In the present study, there was dysfunctional proliferation and differentiation of T cells, as evidenced by the decreased proportions of CD3^+^ T cells, CD3^+^CD4^+^ T cells and CD3^+^CD8^+^ T cells, which might be related to host immunosuppression. In addition, after *G. parasuis* challenge, the piglets presented B-cell dysfunction, as evidenced by the decreased proportion of CD3^–^CD21^+^ B cells.

T-cell exhaustion is characterized by the upregulation of immune checkpoint molecules, a phenomenon that reduces immunopotency [36]. Previous research reported that naïve or immune virus-specific donor CD8^+^ T cells were exhausted after transfusion into carrier recipients in a mouse model of persistent infection with lymphocytic choriomeningitis virus and that co-transfusion of immune CD4^+^ T cells prevented exhaustion of immune CD8^+^ T cells [37]. PD-1 overexpression has been reported to contribute to immune system avoidance in different cancers [38]. PD-1 and PD-L1 are closely related to the progression of human cancers and are promising biomarkers for cancer therapy [39]. The PD-1 signalling pathway plays an important role in immune checkpoint regulation, and upregulated PD-L1 on tumour cells can result in T-cell exhaustion and immune evasion [40]. PD-1 signalling also plays key roles in regulating the magnitude and duration of T-cell activation, and PD-1 can mediate the inhibition of T-cell activation [41]. Tumour cells frequently upregulate PD-L1 to facilitate their escape from phagocytosis by macrophages [42]. In addition, PD-1 is related to increased susceptibility to infections [43]. Cytokine expression levels are important indicators of the function and differentiation of T cells [44]. IL-2 regulates T-cell proliferation, activation and differentiation [45]. Previous research reported that PD-1 suppresses IL-2 production, leading to CD8^+^ T-cell anergy [46]. IL-8 induces M2 macrophage polarisation and inhibits CD8^+^ T-cell infiltration, contributing to the immunosuppressive microenvironment in colorectal cancer [47]. IL-2 and IL-8 were significantly decreased in the blood of *G. parasuis*-challenged piglets, findings similar to those of a study of sepsis-induced immunosuppression [48]. Moreover, *G. parasuis*-challenged piglets showed increased PD-L1 expression in the aorta and a decreased proportion of CD3^+^ T cells in the blood and spleen, consistent with the findings of a previous study [33]. These changes might be an important mechanism by which *G. parasuis* induces host immunosuppression.

Previous studies have reported that PD-1/PD-L1-targeted immunotherapy is a potential approach for treating colon and ovarian cancers [49, 50]. Blocking the interaction between PD-1 and PD-L1 enhances the T-cell response and mediates antitumour activity [51]. Thus, targeting the PD-1/PD-L1 axis might represent a promising therapeutic strategy to control *G. parasuis*-induced host immunosuppression. BMS-1, a PD-L1/PD-1 inhibitor, activates the T-cell–mediated antitumour immune response [52]. In the present study, BMS-1 inhibited PD-1/PD-L1 axis activation and increased the proportions of CD3^+^ T cells, CD3^+^CD4^+^ T cells and CD3^+^CD8^+^ T cells in the blood and splenocytes. Thus, we speculate that BMS-1 blocks the binding of PD-1 and PD-L1, contributing to T-cell differentiation and thereby alleviating host immunosuppression. Levamisole is an immune enhancer that increases immune function in mice with cyclophosphamide-induced immunosuppression [53] and improves the phagocytic functions of monocytes/macrophages and immunomodulatory functions in immunosuppressed mice [54]. Thus, levamisole was used as a positive control in this study. Some traditional Chinese medicines have been reported to enhance the host immune response and to relieve immunosuppression. Korean ginseng berry polysaccharide enhanced the immunomodulatory activities of peritoneal macrophages in mice with cyclophosphamide-induced immunosuppression [55]. Xuanfei baidu decoction, a traditional Chinese medicine formulation, plays a key role in protection against immunosuppression in cyclophosphamide-treated mice [56]. In the present study, baicalin increased the proportion of CD3^+^ T cells, a change that helped alleviate *G. parasuis*-induced host immunosuppression. Based on these findings, baicalin could represent a potential drug candidate for treating *G. parasuis* infection.

Activation of the PI3K/Akt/mTOR pathway is involved in renal cell carcinoma resistance [57]. ERBB3 modulates PI3K/Akt/mTOR pathway activation to alter the epithelial–mesenchymal transition in cervical cancer [58]. Targeted disruption of the PI3K/Akt/mTOR signalling pathway promotes growth inhibition in oral cancer cells [59]. Insulin promotes PD-L1 production and transport in colon cancer stem cells via PI3K/Akt/mTOR signalling [60]. In addition, the PD-1/PD-L1 axis regulates the PI3K/Akt/mTOR pathway, which is involved in the immunosuppressive tumour microenvironment [31]. The PI3K/Akt/mTOR pathway is a potential target for anti-SARS-CoV-2 therapy [61]. The present study showed that baicalin could inhibit PI3K/Akt/mTOR activation, indicating that the PI3K/Akt/mTOR pathway might be a potential target for controlling *G. parasuis* infection.

Taken together, the results showed that baicalin, levamisole and BMS-1 inhibited the PD-1/PD-L1 axis and TIM-3 and activated the PI3K/Akt/mTOR and RAS/MEK/ERK pathways; enhanced the proportions of CD3^+^ T cells, CD3^+^CD4^+^ T cells and CD3^+^CD8^+^ T cells in the blood and splenocytes; attenuated IL-1β, IL-10, IL-18, TNF-α and IFN-γ expression; and prevented tissue damage caused by *G. parasuis* infection. Overall, these treatments provided substantial protection against *G. parasuis* challenge. Baicalin, levamisole and BMS-1 might represent new potential drug candidates to control *G. parasuis* infection and other immunosuppressive diseases.

## Supplementary Information

Additional file 1. **Flow cytometry gating strategy.** A: Gating cell population; B: Excluding adherent cells; C: Gating positive range by single staining; D: Detecting results by double staining.

Additional file 2. **Plots of CD3**^**+**^** T-cell proportions in the splenocyte population and blood.** A1–A7: CD3^+^ T-cell proportions in the splenocyte population; B1–B7: CD3^+^ T-cell proportions in the blood after 24 h; C1–C7: CD3^+^ T-cell proportions in the blood after 48 h.

Additional file 3. **Plots of the proportions of CD3**^**+**^**CD4**^**+**^** T cells in the splenocyte population and blood.** A1–A7: CD3^+^CD4^+^ T-cell proportion of splenocytes; B1–B7: CD3^+^ CD4^+^ T-cell proportion of blood after 24 h; C1–C7: CD3^+^ CD4^+^ T-cell proportion of blood after 48 h.

Additional file 4. **Plots of the proportions of CD3**^**+**^**CD8**^**+**^** T cells in the splenocyte population and blood.** A1–A7: CD3^+^ CD8^+^ T-cell proportions in the splenocyte population; B1–B7: CD3^+^ CD8^+^ T-cell proportions in the blood after 24 h; C1–C7: CD3^+^ CD8^+^ T-cell proportions in the blood after 48 h.

Additional file 5. **Plots of the proportion of CD3**^**–**^**CD21**^**+**^** B cells in the splenocyte population.** A: the control group; B: the infection group; C: the BMS-1 group; D: the levamisole group; E: the 25 mg/kg baicalin group; F: the 50 mg/kg baicalin group; G: the 100 mg/kg baicalin group; BA: baicalin.

## Data Availability

The data supporting the conclusions of this article are included within the article. Additional data used and/or analysed during the current study are available from the corresponding author upon reasonable request.
