# Correction: Baicalin attenuates PD-1/PD-L1 axis-induced immunosuppression in piglets challenged with *Glaesserella parasuis* by inhibiting the PI3K/ Akt/mTOR and RAS/MEK/ERK signalling pathways

**DOI:** 10.1186/s13567-024-01375-x

**Published:** 2024-09-30

**Authors:** Shulin Fu, Jingyang Li, Jiarui You, Siyu Liu, Qiaoli Dong, Yunjian Fu, Ronghui Luo, Yamin Sun, Xinyue Tian, Wei Liu, Jingyi Zhang, Yu Ding, Yitian Zhang, Wutao Wang, Ling Guo, Yinsheng Qiu

**Affiliations:** 1 Hubei Key Laboratory of Animal Nutrition and Feed Science, Wuhan Polytechnic University, Wuhan, 430023 China; 2 Hubei Collaborative Innovation Center for Animal Nutrition and Feed Safety, Wuhan, 430023 China; 3 School of Animal Science and Nutritional Engineering, Wuhan Polytechnic University, Wuhan, 430023 China

**Correction: Veterinary Research (2024) 55:95** 10.1186/s13567-024-01355-1

Following publication of the original article [1], the authors identified an error in Figure 11. The images labelled 50 mg/kg BA and 100 mg/kg BA were inadvertently duplicated.

The original article has been corrected.

## References

[1] Fu S, Li J, You J, Liu S, Dong Q, Fu Y, Luo R, Sun Y, Tian X, Liu W, Zhang J (2024) Baicalin attenuates PD-1/PD-L1 axis-induced immunosuppression in piglets challenged with *Glaesserella parasuis* by inhibiting the PI3K/Akt/mTOR and RAS/MEK/ERK signalling pathways. Vet Res 55:95. 10.1186/s13567-024-01355-1 39075562 10.1186/s13567-024-01355-1 PMC11285455

