# Supplementary figures and images for: Baicalin attenuates PD-1/PD-L1 axis-induced immunosuppression in piglets challenged with Glaesserella parasuis by inhibiting the PI3K/Akt/mTOR and RAS/MEK/ERK signalling pathways
Source: Vet Res. 2024 Jul 29;55:95. doi: 10.1186/s13567-024-01355-1 (PMC11285455; doi:10.1186/s13567-024-01355-1)

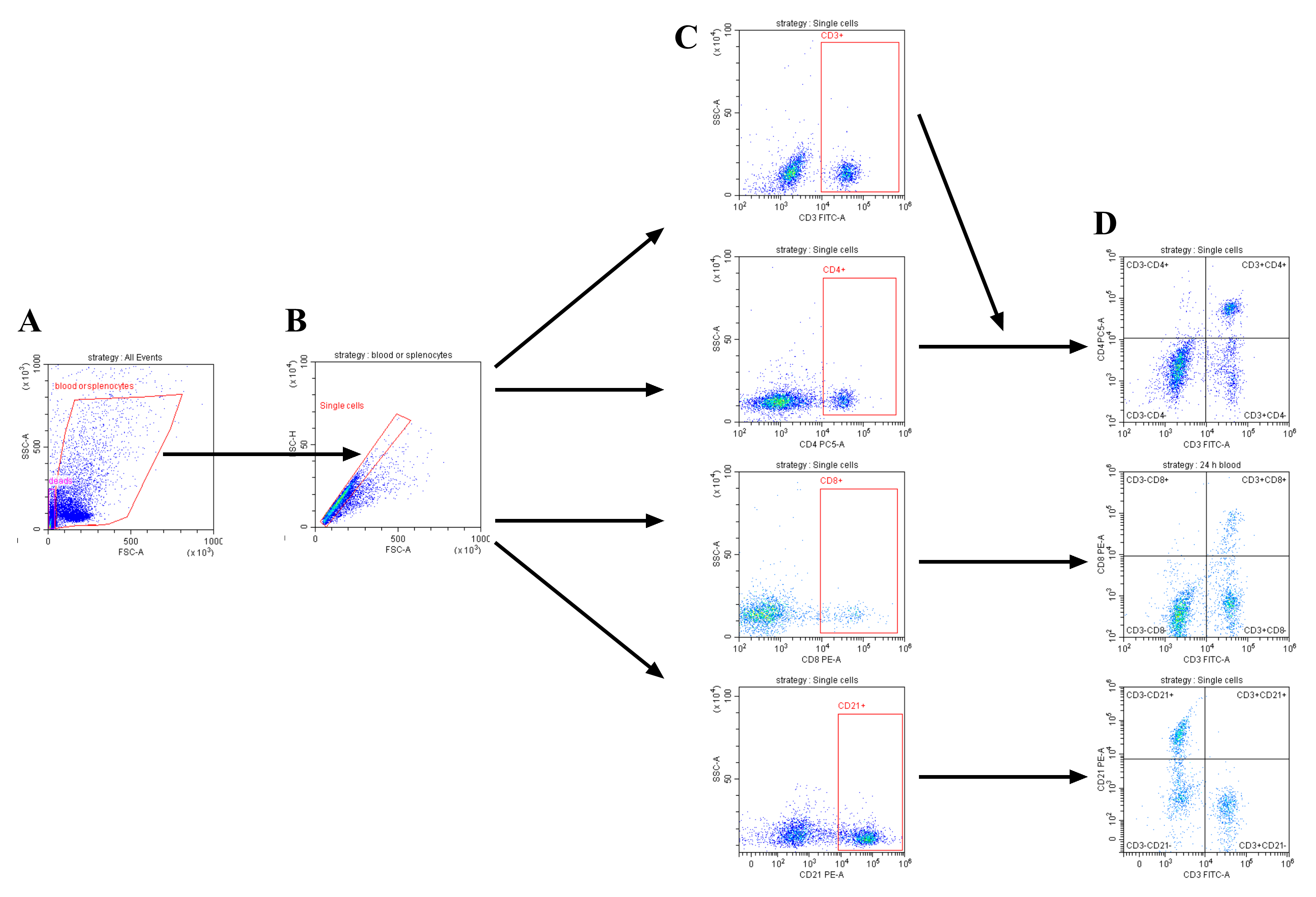

Supplement: Supplementary file 1 — Additional file 1. Flow cytometry gating strategy. A: Gating cell population; B: Excluding adherent cells; C: Gating positive range by single staining; D: Detecting results by double staining. [file 13567_2024_1355_MOESM1_ESM.tif]

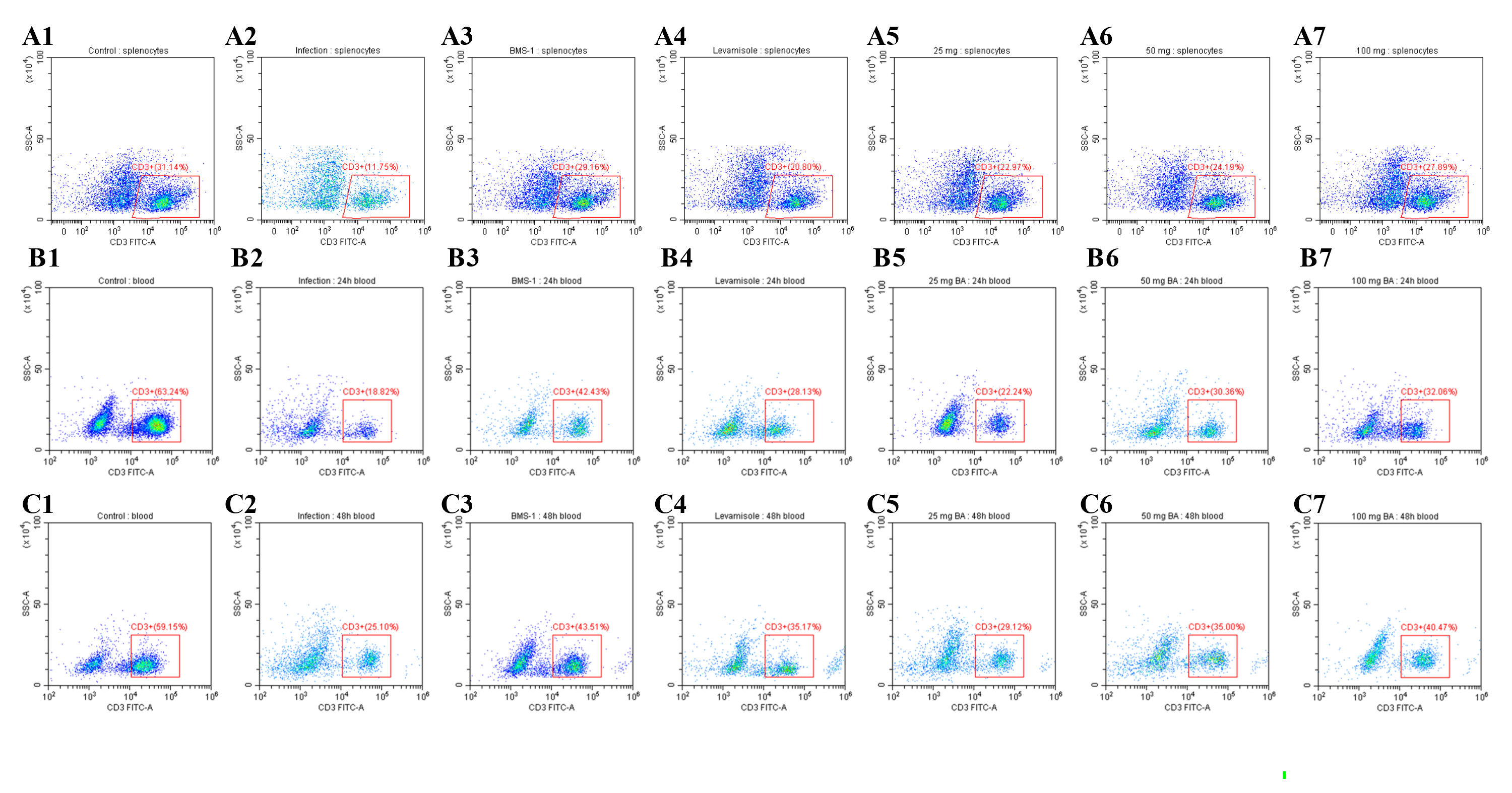

Supplement: Supplementary file 2 — Additional file 2. Plots of CD3+ T-cell proportions in the splenocyte population and blood. A1–A7: CD3+ T-cell proportions in the splenocyte population; B1–B7: CD3+ T-cell proportions in the blood after 24 h; C1–C7: CD3+ T-cell proportions in the blood after 48 h. [file 13567_2024_1355_MOESM2_ESM.tif]

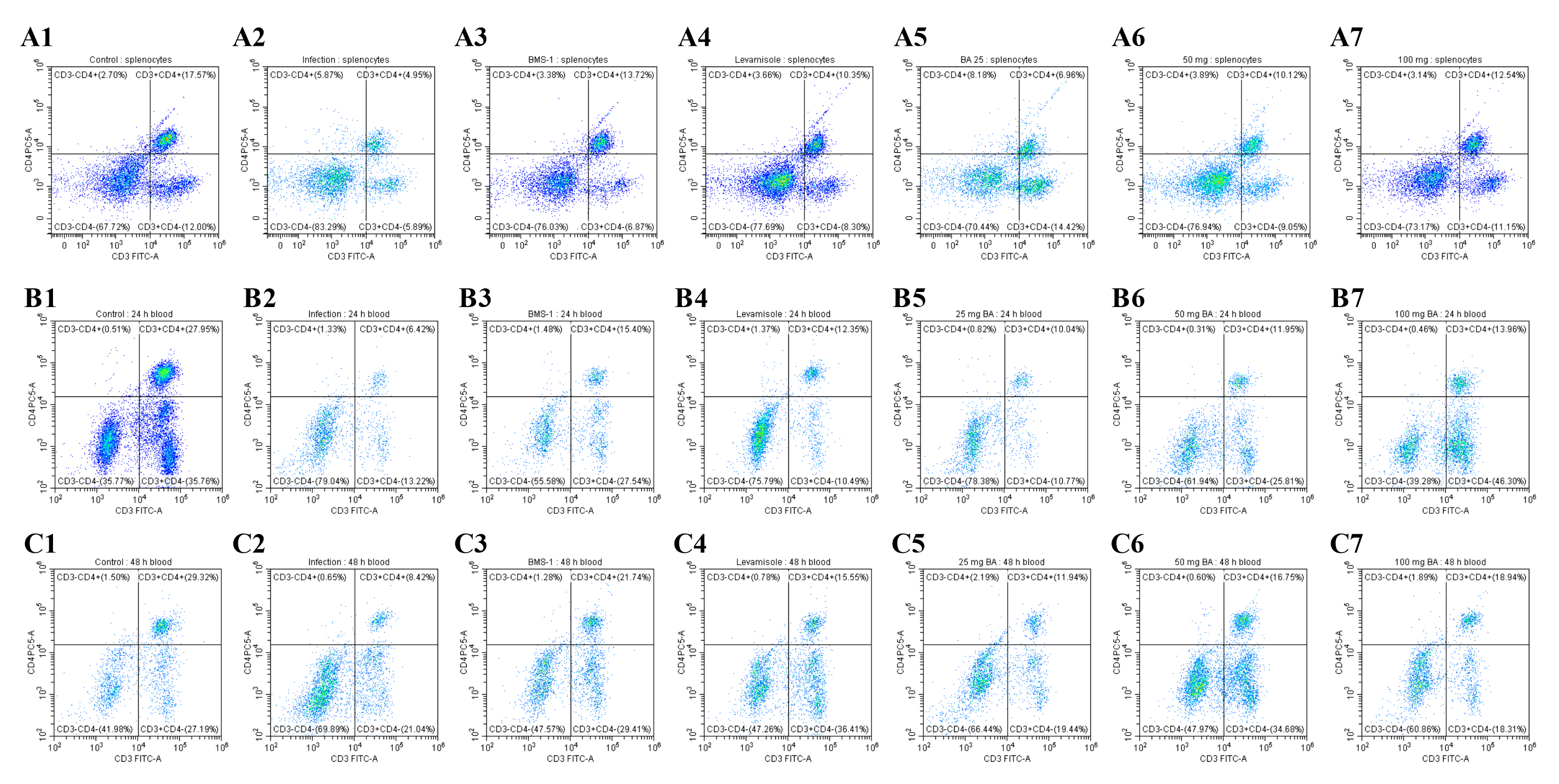

Supplement: Supplementary file 3 — Additional file 3. Plots of the proportions of CD3+CD4+ T cells in the splenocyte population and blood. A1–A7: CD3+CD4+ T-cell proportion of splenocytes; B1–B7: CD3+ CD4+ T-cell proportion of blood after 24 h; C1–C7: CD3+ CD4+ T-cell proportion of blood after 48 h. [file 13567_2024_1355_MOESM3_ESM.tif]

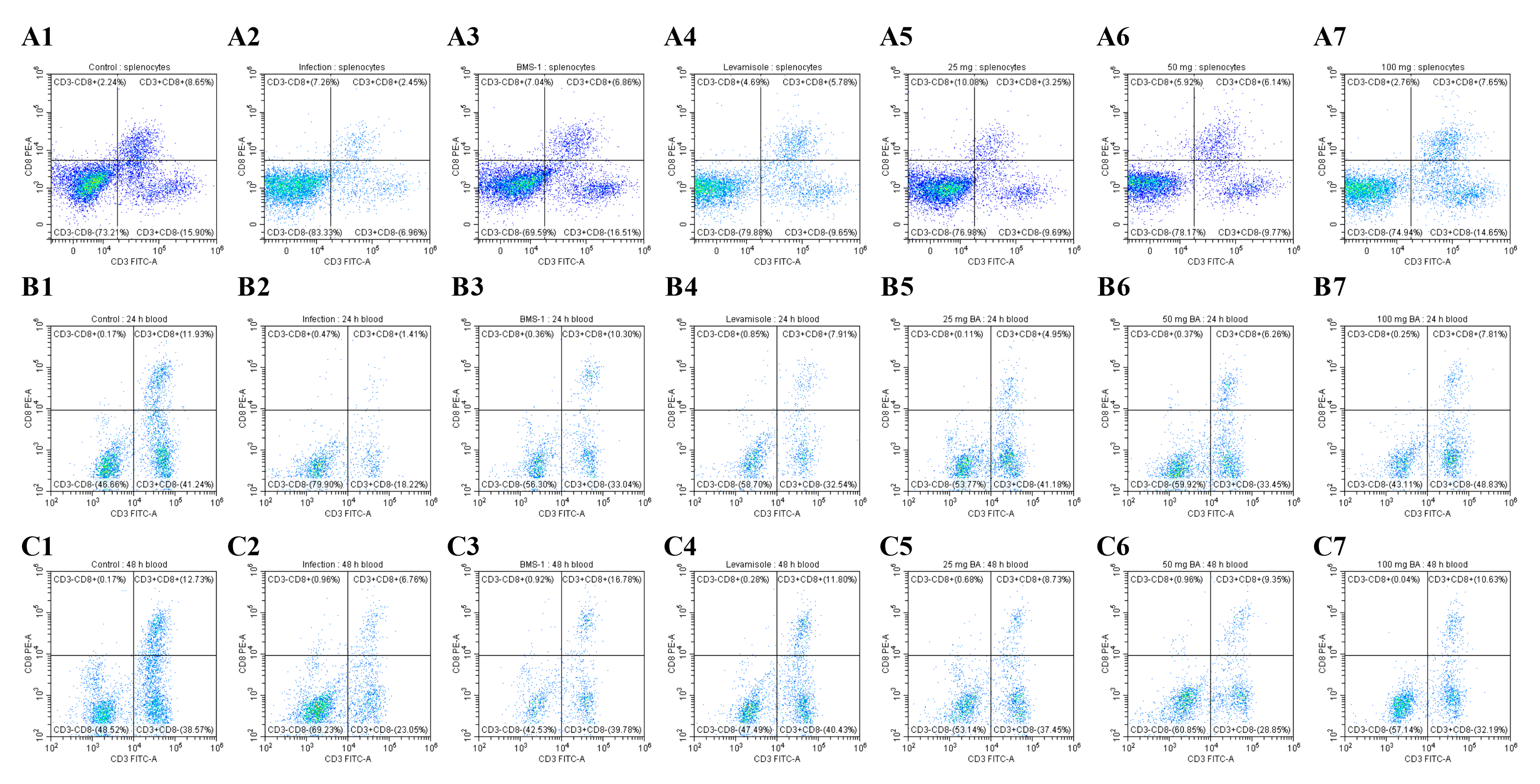

Supplement: Supplementary file 4 — Additional file 4. Plots of the proportions of CD3+CD8+ T cells in the splenocyte population and blood. A1–A7: CD3+ CD8+ T-cell proportions in the splenocyte population; B1–B7: CD3+ CD8+ T-cell proportions in the blood after 24 h; C1–C7: CD3+ CD8+ T-cell proportions in the blood after 48 h. [file 13567_2024_1355_MOESM4_ESM.tif]

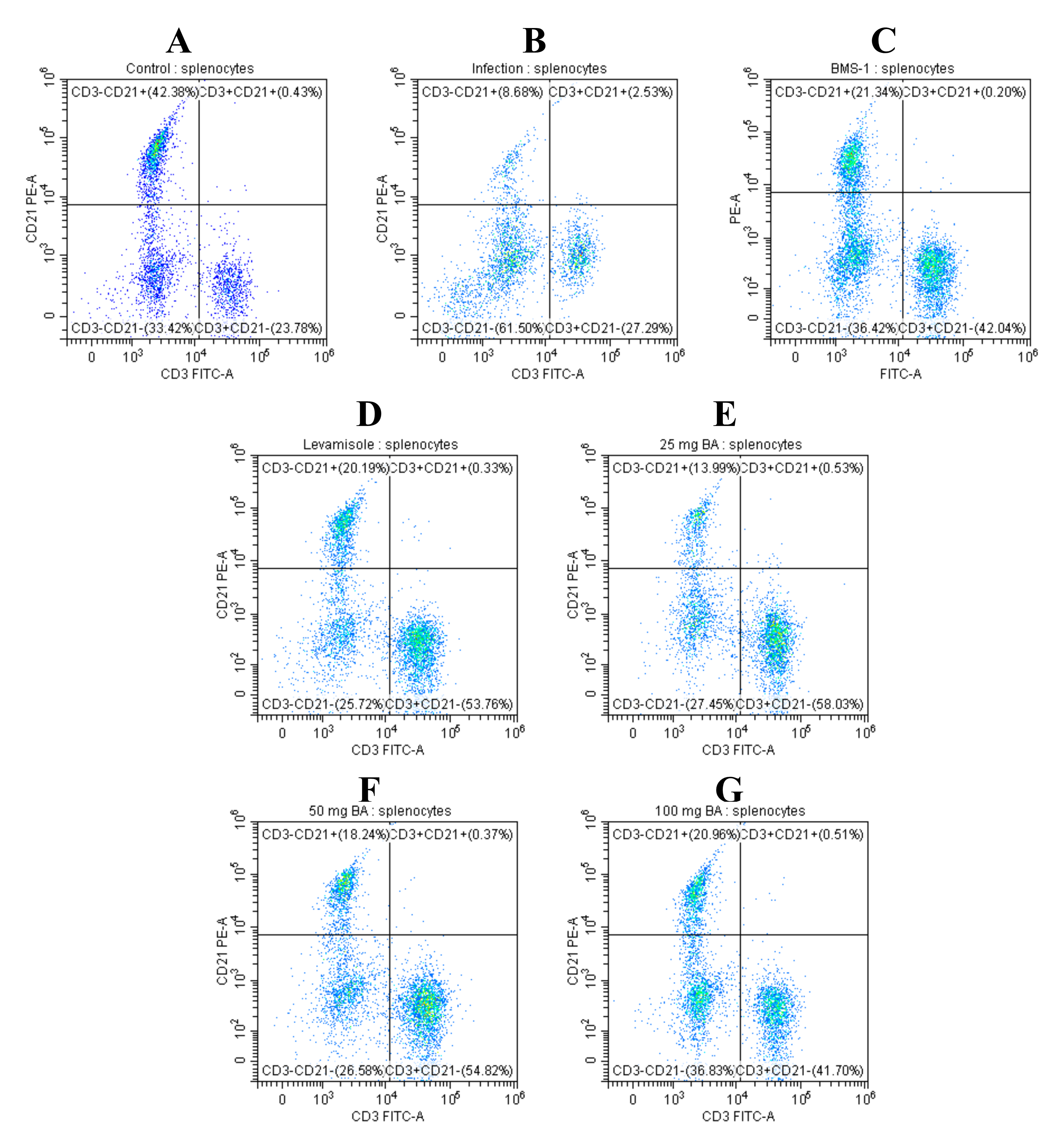

Supplement: Supplementary file 5 — Additional file 5. Plots of the proportion of CD3–CD21+ B cells in the splenocyte population. A: the control group; B: the infection group; C: the BMS-1 group; D: the levamisole group; E: the 25 mg/kg baicalin group; F: the 50 mg/kg baicalin group; G: the 100 mg/kg baicalin group; BA: baicalin. [file 13567_2024_1355_MOESM5_ESM.tif]
